# The effects of internal representations on performance and fluidity in a motor task

**DOI:** 10.1007/s00426-023-01912-x

**Published:** 2024-01-12

**Authors:** Oliver R. Runswick, Hettie Roebuck

**Affiliations:** 1 Department of Psychology, Institute of Psychiatry, Psychology and Neuroscience, King’s College London, Guy’s Campus, London, SE1 1UL UK; 2 School of Psychology, University of Derby, Kedleston Road, Derby, DE22 1GB UK

## Abstract

Individuals can differ in the mode in which they experience conscious thought. These differences in visualisation and verbalisation can also be evident during motor control. The Internal Representation Questionnaire (IRQ) was developed to measure propensity to engage certain types of representations, but its ability to predict motor control and links to reinvestment and motor imagery have not been tested. 159 included participants completed the IRQ, movement specific reinvestment scale (MSRS), and a novel online motor task before and after a period of practice. Results showed that the IRQ Verbal and Orthographic factors were significant predictors of scores on the MSRS. The IRQ factor of Manipulational Representations predicted motor performance both before and after practice. The fluidity of executed movements were predicted by the IRQ verbalisation factor where higher propensity to verbalise was associated with higher levels of jitter, but only after a period of practice. Results suggest there may be some informative conceptual overlap between internal verbalisations and reinvestment and that the propensity to manipulate internal representations may be predictive of motor performance in new tasks. The IRQ has potential to be a valuable tool for predicting motor performance.

## Introduction

Whether it is remembering an experience you had with your friends, or solving a problem at work, humans can experience the representation of these thoughts in different ways (Roebuck & Lupyan, 2020). Individual differences in experience of conscious thought (phenomenology) were first studied in 1880 when Sir Francis Galton published the results of work on individuals’ abilities to visually imagine different forms of information (Galton, 1880a, 1880b). It is, however, not just in visual imagery that individuals can differ in their experiences of thought. An aspect of phenomenology that is less studied, but linked with cognitive processes, is the tendency to experience thoughts in the form of language (internal verbalisation; Alderson-Day & Fernyhough, 2015a, 2015b; Hurlburt et al., 2013; Lupyan, 2016; Lupyan & Bergen, 2016). Some individuals report having a propensity to use an inner voice outside of communicating, while others have a propensity to create visual images (Roebuck & Lupyan, 2020). An individual engaging in verbalisation or visualisation does not just affect cognitive processes, but also the control of movements (Beilock & Carr, 2001; Hardwick et al., 2018).

Skilled motor performance is a unique interaction of conscious and automatic control (Pacherie & Mylopoulos, 2020). However, in the context of motor control, a high level of conscious verbal processing can have negative effects on performance and skilled movement can often be characterized by low levels of conscious control (Deeny et al., 2003; Masters, 1992). The literature investigating this has used measures of an individual’s ability to verbalise aspects of a motor skill to investigate possible mechanisms leading to poor performance and is applicable to fields such as fear of falling in the elderly (Young et al., 2020), and the performance of surgeons (Malhotra et al., 2014) and sports people (Malhotra et al., 2015). For example, in a continuous walking task, controlling elements of movement execution such as, “extend leg, place foot in safe location, transfer weight”, can decrease the efficiency of gait and increase the chances of falling (Ellmers & Young, 2018). In a closed sporting task, across four experiments, Beilock and Carr (2001) measured memory processes engaged during golf putting in experts and novices. Results showed impoverished episodic recall (recollection of specific events such as execution of stance, grip, back swing, and follow-through) in experts compared to novices, suggesting a reduction in conscious control, and increased engagement of more efficient procedural (automated and outside of conscious control) memory processes. The findings suggest that a propensity to internally verbalise during a motor task could lead to more novice-like motor performance and less fluid movements.

To conceptualise the negative effects conscious motor control can have on performance, Masters and Maxwell (2008) developed the theory of reinvestment, proposing that differences exist between individuals’ propensities to engage in executive control to regulate behaviours. Conscious control processes can disrupt natural automated control processes (Bellomo et al., 2018), and are more akin to how a novice would perform a task, so can result in a significant breakdown of performance. However, reinvestment only occurs after a movement has been learned. A performer ‘reinvests’ facts that can be consciously accessed and verbalized from the learning process (declarative memory structures; Masters & Maxwell, 2008). They oppose reinvestment to the process of engaging procedural memory (more automated processes) for skill execution. Reinvestment does not predict individual differences in motor learning, but instead individual differences in how movements are controlled during performance under pressure after they have been learned or practiced (Malhotra et al., 2014, 2015).

To measure this individual propensity to reinvest during motor control, Masters et al., (2005 developed the movement specific reinvestment scale (MSRS; Masters et al., 2005). The scale consists of ten questions relating to engagement of executive control during movements, which are in turn divided into two sub-scales: conscious motor processing (CMP), referring to the individual’s propensity to consciously control movements, and movement self-consciousness (MSC) referring to an individual’s propensity to monitor the style of their movement (Ling et al., 2016; Uiga et al., 2018). The scale has been widely applied in a range of literature that has investigated reinvestment across populations. This work has shown considerable support for the theory of reinvestment (Bellomo et al., 2018; Kinrade et al., 2015; Malhotra et al., 2015; Uiga et al., 2018; Weiss, 2011; Zhu et al., 2011). For example, when investigating motor control processes in the elderly, Uiga et al. (2018) showed relationships between MSRS scores, and kinematics associated with gait control. Despite the importance of understanding these individual motor control processes for supporting motor learning and performance, and the potential conceptual overlap with internal verbalisation, there have been no investigations that attempt to bridge the phenomenology literature with conscious motor control.

Propensity to engage conscious control in action is not the only way internal representations have been indirectly investigated in the motor control literature; internal visualisation has also been widely investigated in this context (Roberts et al., 2008). It is widely suggested that ‘motor imagery’, “the mental execution of a movement without any movement or peripheral muscle activation” (Mulder, 2007, p. 1265) is beneficial to motor learning and control. Hardwick et al. (2018) conducted a meta-analysis of 303 experiments on 4902 participants. The authors concluded that motor imagery activates similar subcortical networks as executing the movement itself. Other empirical work has linked this activation to improvements in motor performance (Kim et al., 2017; Romano-Smith et al., 2018). Despite the value in establishing predictive relationships between visualisation and verbalisation, and motor performance, most of the literature has only investigated one type of representation in isolation. This approach may not reveal the full nature of the relationship between internal representations (i.e., interactions between verbalisation and visualisation) and motor control.

To consolidate the measurement of internal verbalisation with other individual differences in experiences of thought (e.g., visualisation), Roebuck and Lupyan (2020) developed the internal representation questionnaire (IRQ). The IRQ built on a variety of previously constructed questionnaires that measured visualisation and verbalisation and other forms of auditory or tactile imagery (Alderson-Day et al., 2018; Brinthaupt et al., 2009; Kirby et al., 1988). By including these items in a single instrument, the IRQ focuses on an individual’s *propensity* to engage in different types of representation rather than an individual’s *ability* to do so. The development of the IRQ resulted in a 36-statement instrument grouped into four factors; visual imagery (IRQ Visual), internal verbalisation (IRQ Verbal), orthographic imagery (referring to the visualisation of language; IRQ Orthographic) and manipulational representation (referring to the propensity to manipulate representations; IRQ Manipulation). These are factors that have potential to predict individual differences in motor control processes. However, most of the research that has investigated representations and motor control separately investigated an individual’s ability to visualise actions (Roberts et al., 2008), the use of visual and verbal instruction (Al-abood et al., 2001) or the propensity of an individual to engage in verbalisation during conscious motor control (Beilock & Carr, 2001; Hoskens et al., 2020; Masters & Maxwell, 2008; Uiga et al., 2018), but not at these forms of representations together. If an increased propensity for visualisation can be beneficial to motor performance (Hardwick et al., 2018), and internal verbalisations can have negative effects (Beilock & Carr, 2001), a tool that can combine measurement of an individual’s propensity to engage in verbalised control during movement execution with measures of visual imagery ability could prove a powerful tool in predicting motor performance. The IRQ then could prove highly valuable for applications including stroke rehab (Johnson et al., 2013), training programmes for demanding professions (e.g., sports; Steenbergen et al., 2010), and even predicting fall likelihood in the elderly (Uiga et al., 2018).

One of the practical challenges in extending this literature is the recruitment of sufficient participants to execute motor tasks and establish predictive relationships between internal representations and motor performance. This is combined with the task fragmentation that is present in motor learning literature where the same tasks are rarely used across studies to build knowledge (Ranganathan et al., 2021). However, recent developments in online data collection platforms offer a possible solution to both issues. Tsay et al. (2021) investigated the validity and viability of conducting sensory motor learning studies using online platforms and crowdsourcing for recruitment. Results showed that data closely corresponded to data collected using the same procedures in the lab. One method of collecting detailed data on movement execution that has been popular in the lab is mouse tracking (Cranford & Moss, 2018; Kieslich et al., 2019; Rheem et al., 2018). This process records coordinates of the participant’s cursor during task execution, a feature that is now available on the online data collection platform ‘Gorilla’ (Anwyl-Irvine et al., 2020). This affords the opportunity to expand motor control research to larger numbers of people, test predictive relationships between internal representations and motor performance in a model task and use mouse tracking to measure variables such as ‘jitter’ that captures the fluidity of movements (Kieslich et al., 2019; Wulff et al, 2019).

If conscious and verbalisable control of movements negatively affect motor performance (Beilock & Carr, 2001; Beilock et al., 2002), but motor imagery can be beneficial (Kim et al., 2017; Romano-Smith et al., 2018), it is perhaps strange that the study of how individuals represent their thoughts (internal representation) has generally remained distinct from the study of motor control. There is a need to establish the relationship between the measurement of internal representations in phenomenology literature and measures used in motor control work. This would help to develop a combined understanding of how individual differences in internal representations can affect motor control processes. To address this need, participants completed measures of internal representations (IRQ Verbal, Visual, Orthographic, and Manipulation) and reinvestment (Total Reinvestment, CMP, and MSC) and had their motor performance efficiency (speed/accuracy trade off) and jitter of the mouse cursor measured on a novel online continuous, closed-loop precision motor task before and after a period of practice. We predicted that a higher propensity to internally verbalise (IRQ Verbal) will be positively associated with an individual’s propensity to engage conscious processing during movement and therefore predict indexes of (i) Total Reinvestment, (ii) CMP and (iii) MSC scores. IRQ Verbal, Total Reinvestment will be negatively associated with motor performance efficiency and but only after the task is learned. A higher propensity to visualise a task will be positively associated with motor performance efficiency both before and after the task has been learned. IRQ Verbal, Total Reinvestment will be associated with higher levels of jitter in movement execution and but only after the task is learned. A higher propensity to visualise a task will be associated with lower levels of jitter both before and after the task has been learned.

## Method

### Participants

An a priori sample size calculation for a fixed model linear multiple regression was conducted using G*Power (Faul et al., 2007). Due to the novelty of this design and, therefore, lack of effect sizes in previous literature or pilot work, we used the smallest effect size of interest approach (Lakens, 2021). We used a small effect size (*f* = 0.085), alpha of 0.5, and, due to the historically low power in work in this area, a power of 0.95. This resulted in a required sample of 155 participants.

To account for drop out and failing attention checks, a total of 246 participants were recruited, via the online recruitment platform Prolific (prolific.co). For analysis a total of 159 participants were retained following exclusion criteria. First data were screened for engagement, an important process when collecting data remotely and online (Peer et al., 2021; Tsay et al., 2021). Participants were removed for failing any one of the three attention checks (items in the questionnaire where there was a specified correct response e.g., select the option ‘strongly disagree’), a total of 13 participants were removed for failing any one of these 3 checks. Next, mouse tracking coordinates for all trials in the motor task section of the study were screened for incorrect routes. Participants who executed the wrong route across at least 4/5 trials in a single block through either a lack of engagement or misunderstanding of instructions had their data removed. In total, a further 69 participants were excluded on this basis leaving 159 who completed the entire protocol. Analysis was conducted only on trials where participants went the correct route. Error checking on correctly executed routes revealed participants hit an obstacle or the edge of screen on 8.9% of trials in novel performance and 7.9% in learned performance.

Included participants were 18–64 years old (mean = 35.26, SD = 10.93), 93 female, 65 male, and 1 who did not wish to identify. 158 participants were right hand dominant. Participants were required to participate on a computer with a trackpad, have normal or corrected to normal vision, and report no hand or wrist injuries or coordination difficulties. Prolific has been shown to produce the best quality data compared to other online recruitment tools (Peer et al., 2021) and participants were reimbursed in line with the Prolific fair pay policy. All participants completed informed consent and ethical approval was granted by the University research ethics committee.

### Design

**Fig. 1:**
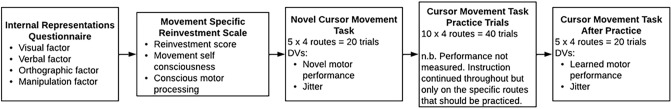
Overview of study and variables collected at each phase

Participants completed five phases of testing (Fig. 1). To test the first hypothesis, the Internal Representation Questionnaire (Roebuck & Lupyan, 2020) was used to investigate internal representations and the Movement Specific Reinvestment Scale (Malhotra et al., 2015; Masters et al., 2005) was used to measure an individual’s overall disposition for conscious control of movement. The novel online cursor movement task measured motor performance efficiency through response time and error rate and fluidity through jitter before and after the participants had an opportunity to practice.

### Materials

*Internal Representations Questionnaire (IRQ)* The IRQ (Roebuck & Lupyan, 2020) was developed to measure individual differences in experiences of moment-to-moment thought. The IRQ was developed predominantly to measure use of language in thought processes and includes a total of 36 questions rated from 1 (strongly disagree) to 5 (strongly agree) that investigate four broader interrelated but unique factors (visual, verbal, orthographic, and manipulational representation). The visual factor (IRQ Visual) includes a set of 10 questions such as ‘I can close my eyes and easily picture a scene I have experienced’ (*r* = 0.78, Cronbach's *α* = 0.86). The verbal factor (IRQ Verbal) includes 12 items related to the use of an inner voice such as ‘I think about problems in my mind in the form of conversations with myself’ (*r* = 0.68, Cronbach's *α* = 0.86). The orthographic factor (IRQ Orthographic) consists of six questions related to the visualisation of language such as ‘when I hear someone talking, I see words written down in my mind’ (*r* = 0.65, Cronbach's *α* = 0.72). The final factor investigates the manipulations of mental representations (IRQ Manipulation) and includes eight questions such as ‘I can easily mentally rotate three-dimensional geometric figures’ (*r* = 0.64, Cronbach's *α* = 0.79). Mean scores were calculated for each factor. Higher scores mean a higher propensity. Attention checks included in the original development of the survey were also included in this study.

*Movement Specific Reinvestment Scale (MSRS)* The MSRS (Masters et al., 2005) is an adapted version of the original reinvestment scale (Masters et al., 1993). It consists of ten questions measuring two constructs. Conscious motor processing (CMP; *r* = 0.76, Cronbach's *α* = 0.71) such as, ‘I am aware of the way my body works when I am carrying out a movement,’ and movement self-consciousness (MSC; *r* = 0.67, Cronbach's *α* = 0.78), such as, ‘I am concerned about my style of moving’ (see Malhotra et al., 2015). Each item is rated on a six-point Likert scale ranging from strongly disagree (1) to strongly agree (6). Here, we calculated a Total Reinvestment Score, a CMP score, and a MSC score by using the mean Likert the score from the relevant questions. A higher score means a higher propensity to reinvest.

**Fig. 2:**
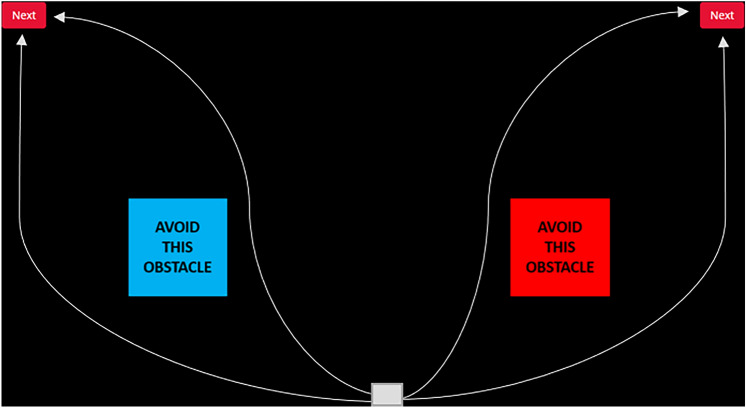
Design of the motor task. The white box represents where the participant’s cursor appeared at the start of the task and the four white lines represent the four possible routes the participants were instructed to take

*Cursor movement task* We developed a bespoke online motor task (Fig. 2) adapted from previous literature that used cursor movement tasks to measure motor control (Cranford & Moss, 2018; Kimura & Nakano, 2019; Maraj et al., 2003). This continuous, closed-loop, precision motor task was chosen as it incorporates the speed accuracy trade off applicable to many areas of motor performance (such as sports; Beilock & Carr, 2001), a continuous element that is present in conscious control work in gait (Ellmers & Young, 2018), and offers enough complexity to potentially require multiple elements of verbalisable control. The task was hosted on the online platform Gorilla Experiment Builder (www.gorilla.sc; Anwyl-Irvine et al., 2020) and involved using the track pad with the non-dominant hand to navigate the cursor on one of four specified routes to click the button as fast as possible without leaving the task area or hitting an obstacle. Participants completed each of the four routes five times with their response times and error rate calculated before being given an opportunity to practice each route ten times. After practice, participants repeated each route five further times. Route orders were counter balanced. Participants were clearly informed when their performance was being measured in the novel and learned conditions and were told the routes they practiced would be checked but performance would not be measured in the practice section.

Participants were given clear instructions about which route to execute on each trial. All instructions for the motor task were written to account for any effects of preference for verbal or visual instruction types (Maraj et al., 2003). For example, *‘Using your non-dominant hand move the cursor UP and OVER THE BLUE OBSTACLE to click the LEFT next button as quickly as you can without hitting an obstacle or leaving the black screen. Your time will start as soon as the next screen appears. Click next when you are ready.’* When a participant clicked ‘next’ the task would appear with the cursor in the set position shown by the white box in Fig. 2. Errors (hitting an obstacle or leaving the screen) and incorrect routes were detected by tracking the normalised coordinates of the cursor and checking against location of the obstacles and screen edge. Incorrect routes did not count as errors and were instead removed from analysis. To calculate performance, response times and error rates were averaged across routes to account for route difficulty. To assess the fluidity of movements jitter was calculated using the mouse tracking coordinates.

### Procedure

Participants were directed via a URL link to online survey platform Qualtrics (Qualtrics, Provo, UT). Here, they received a full information sheet and completed written informed consent. Once they had consented, they completed the IRQ and MRSR on Qualtrics. On completion of the surveys, a link directed them to the Gorilla platform in which they received instructions and completed the novel mouse movement task (five attempts at each of the four routes), practice trials (ten attempts at each of the four routes), and mouse movement task after practice (five attempts at each of the four routes) elements of the study. Upon completion of the learned task, participants received a written debrief including information on data withdrawal.

### Data analysis

*Motor performance* By recording response times and number of errors, a movement performance score was calculated to incorporate the speed-accuracy trade-off in movement execution. The approach outlined by Gredin et al., (2018) was applied by multiplying the number of errors by the response time meaning a lower score shows more efficient movement. To account for participants who make no errors (which would result in a score of zero), an error score was created where no errors received a score of 1 (so their efficiency score would equal their average time) and 0.05 was added to the total error score of every trial with an error. The variable ‘novel task performance’ represents performance across the first 20 trials and ‘learned task performance’ across the last 20 trials.

*Jitter* The number of directional changes on the x axis (x-flips) were calculated for each correct trial. X-flips have been suggested to be an intuitive measure of movement instability, or instantaneous changes in momentary valence (Koop & Johnson, 2013).

*Task engagement* To check improvement in the task during the practice period, and therefore engagement with the task, we conducted a paired samples t-test to compare motor performance during novel and learned phases. The alpha level (*p*) for statistical significance was set at 0.05 and Cohen’s d was used to calculate the effect size.

*Regressions* To test the predictive nature of the relationships forced entry regression models were constructed to address each hypothesis. First, three models tested the first hypothesis and were constructed with the four IRQ factors as predictor variables and Total Reinvestment, CMP and MSC as outcome variables. In further models only, the Total Reinvestment score was included and not CMP or MSC due to strong relationships between factors. The fourth and fifth models tested the second hypothesis and used the four IRQ factors, Total Reinvestment as predictors and Novel Performance and Learned Performance as outcome variables for motor performance, and the final models used ‘jitter’ as the outcome variable. To examine the role of internal representation propensities on the above outcome measures, we used a series of linear regression models were implemented in R’s `lme4` package (Bates et al., 2015). All variables were scale centered.

## Results

### Task engagement

**Fig. 3:**
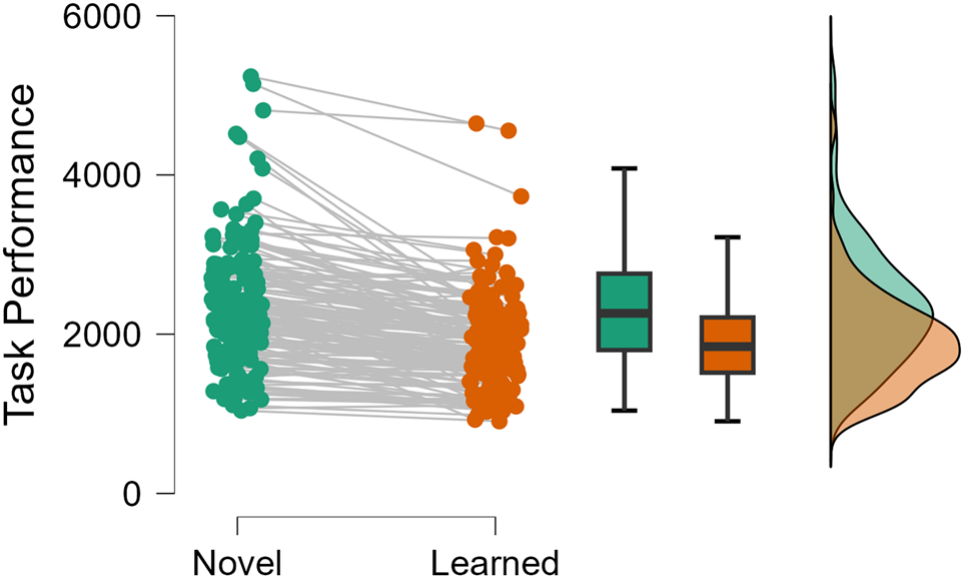
Raincloud plot to show improvements in performance from pre-test to post-test (lower score means more efficient task performance).

Participants performed significantly better on the task after the practice period (mean efficiency score for learned performance = 1903.861 ± 606.141) compared to the novel test phase before they had been able to practice the task (mean efficiency score for novel performance = 2377.351 ± 851.528; *t* = 10.962, *p* < 0.001, *d* = 0.869; Fig. 3).

### IRQ and total reinvestment

**Fig. 4:**
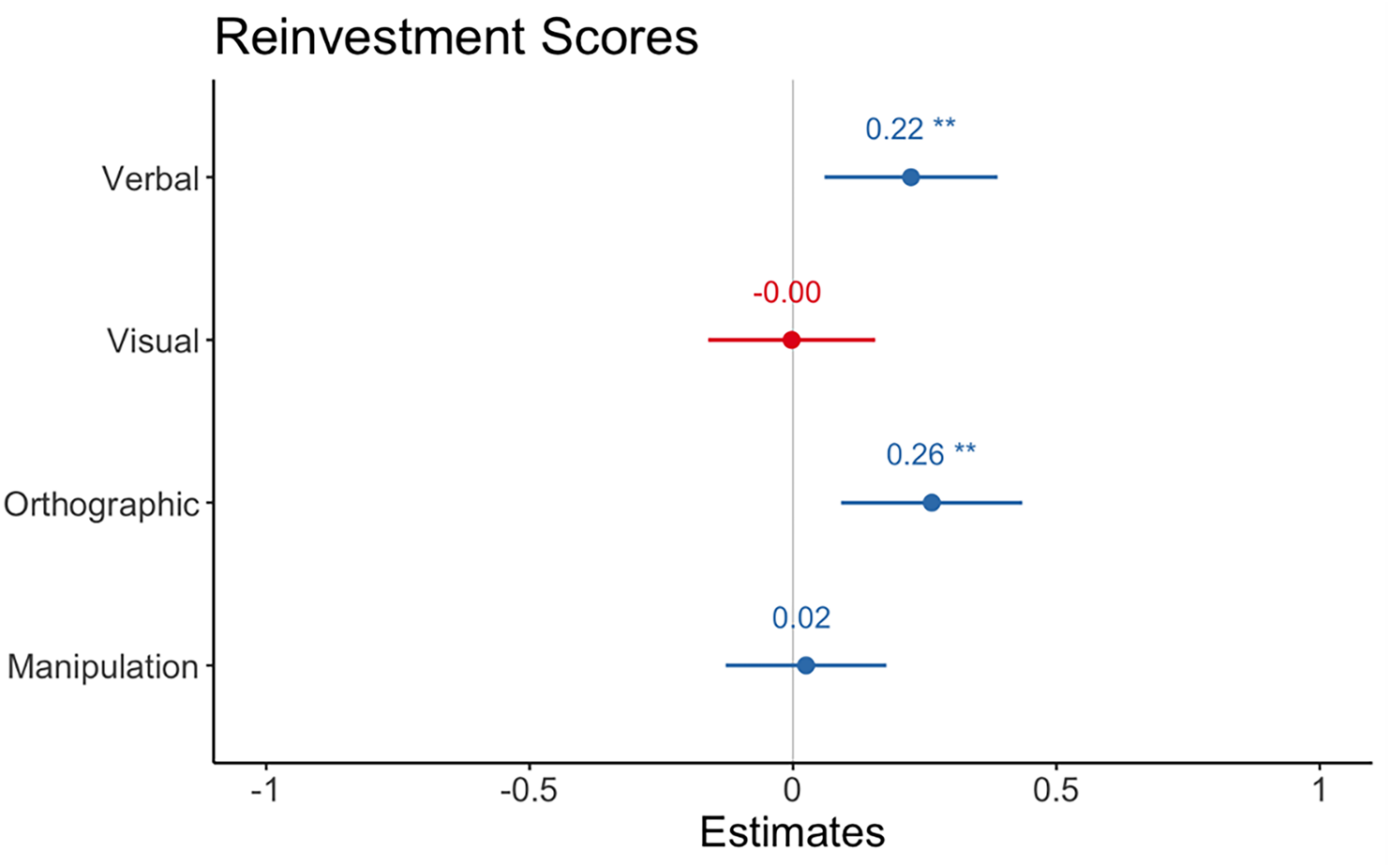
Regression model estimates from linear model for predictors of Reinvestment. Asterix denotes significant effects. Error bars show 95 percent CI

Degree of internal verbalisation predicted total reinvestment, those with a higher propensity to verbalise had higher reinvestment scores *b* = 0.22, 95% CI = [0.06, 0.39], *z* = 2.69, *p* = 0.008. Degree of visual imagery did not predict reinvestment *b* = – 0.002, 95% CI = [– 0.16, 0.16], *z* = – 0.03, *p* = 0.97. Degree of orthographic representations positively predicted reinvestment scores *b* = 0.26, 95% CI = [0.09, 0.44], *z* = 3.03, *p* = 0.003. There was no significant effect of representational manipulation on total reinvestment *b* = 0.02, 95% CI = [– 0.13, 0.18], *z* = 0.32, *p* = 0.75 (see Fig. 4).

### IRQ and movement self-consciousness (MSC)

The same pattern of effects is observed for the sub-scale for movement self-consciousness. Degree of internal verbalisation predicted MSC, those with a higher propensity to verbalise had higher MSC scores *b* = 0.32, 95% CI = [0.12, 0.53], *z* = 3.22, *p* = 0.002. Degree of visual imagery did not predict MSC *b* = – 0.05, 95% CI = [– 0.25, 0.14], *z* = – 0.55, *p* = 0.58. Degree of orthographic representations positively predicted MSC scores *b* = 0.34, 95% CI = [0.13, 0.55], *z* = 3.21, *p* = 0.002. There was no significant effect of representational manipulation on MSC *b* = – 0.08, 95% CI = [– 0.27, 0.11], *z* = – 0.83, *p* = 0.40.

### IRQ and conscious motor processing (CMP)

Internal representations did not predict CMP. No significant effects were observed for verbal *b* = 0.11, 95% CI = [– 0.03, 0.26], *z* = 1.60, *p* = 0.11.), visual b = 0.06, 95% CI = [– 0.07, 0.21], *z* = 0.95, *p* = 0.34, orthographic *b* = 0.12, 95% CI = [-0.03, 0.27], *z* = 1.61, p = 0.11, or representational manipulation representations b = 0.09, 95% CI = [– 0.04, 0.23],], *z* = 1.41, *p* = 0.16.

### IRQ, reinvestment, and novel task efficiency

**Fig. 5:**
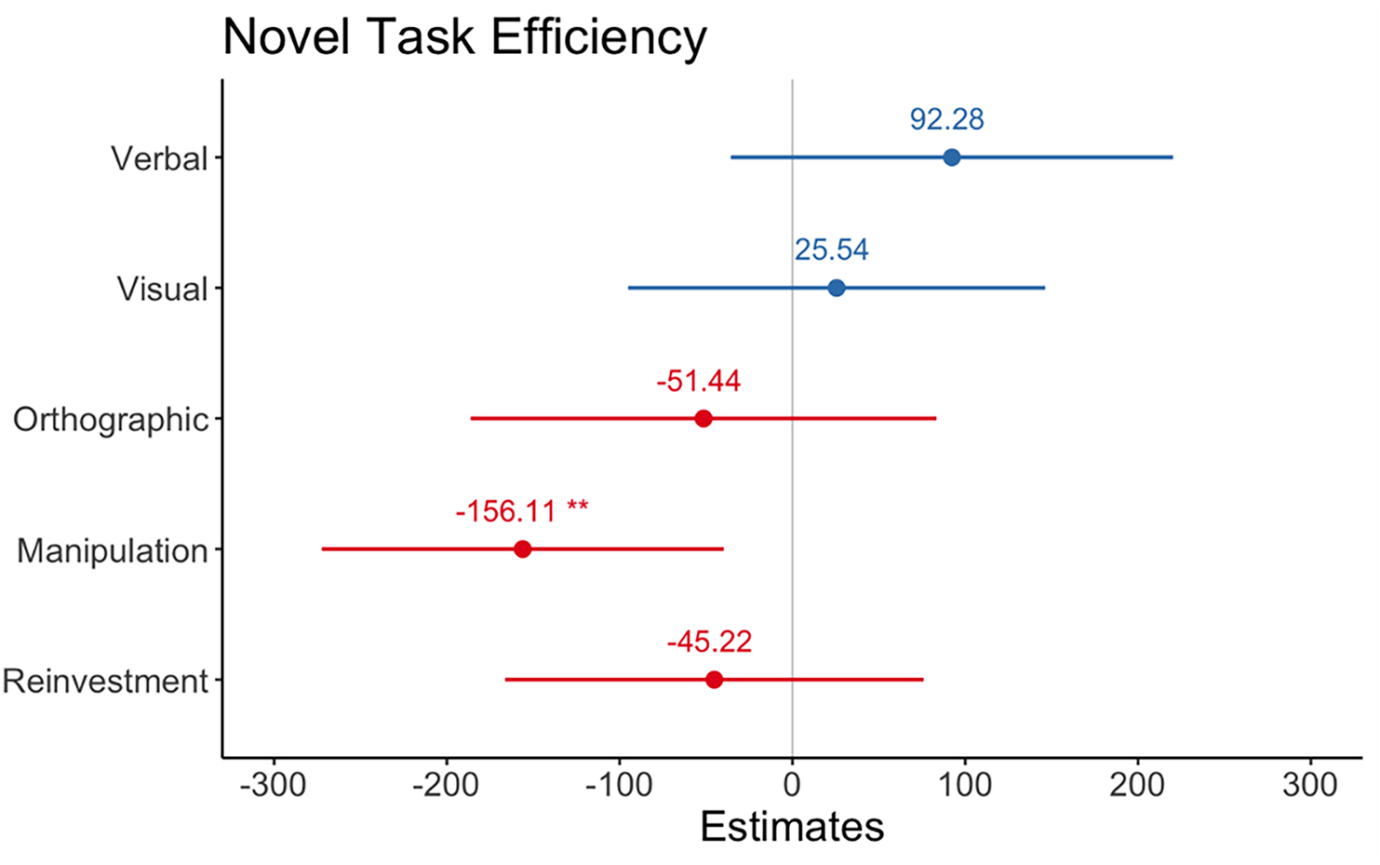
Regression model estimates from linear model for predictors of Novel Task Efficiency. Asterix denotes significant effects. Error bars show 95 percent CI

Degree of representational manipulation predicted performance when the task was novel, those who reported higher representational manipulation were more efficient at performing the task *b* = – 0.156.11, 95% CI = [– 272.42, – 39.79], *z* = – 2.65, *p* = 0.009. No significant effects were observed for Verbal *b* = 92.28, 95% CI = [– 0.03, 0.26], *z* = 1.42, *p* = 0.16., Visual b = 25.54, 95% CI = [– 95.15, 0.146.25], z = 0.42, *p* = 0.68 or Orthographic Representations b = 51.44, 95% CI = [– 0.186.26, 0.83.37], z = – 0.75, *p* = 0.45. There was also no significant effect of Total Reinvestment *b* = 45.22, 95% CI = [– 166.25, 0.75.79], *z* = – 0.73, *p* = 0.46 (see Fig. 5).

### IRQ, reinvestment, and learned task efficiency

**Fig. 6:**
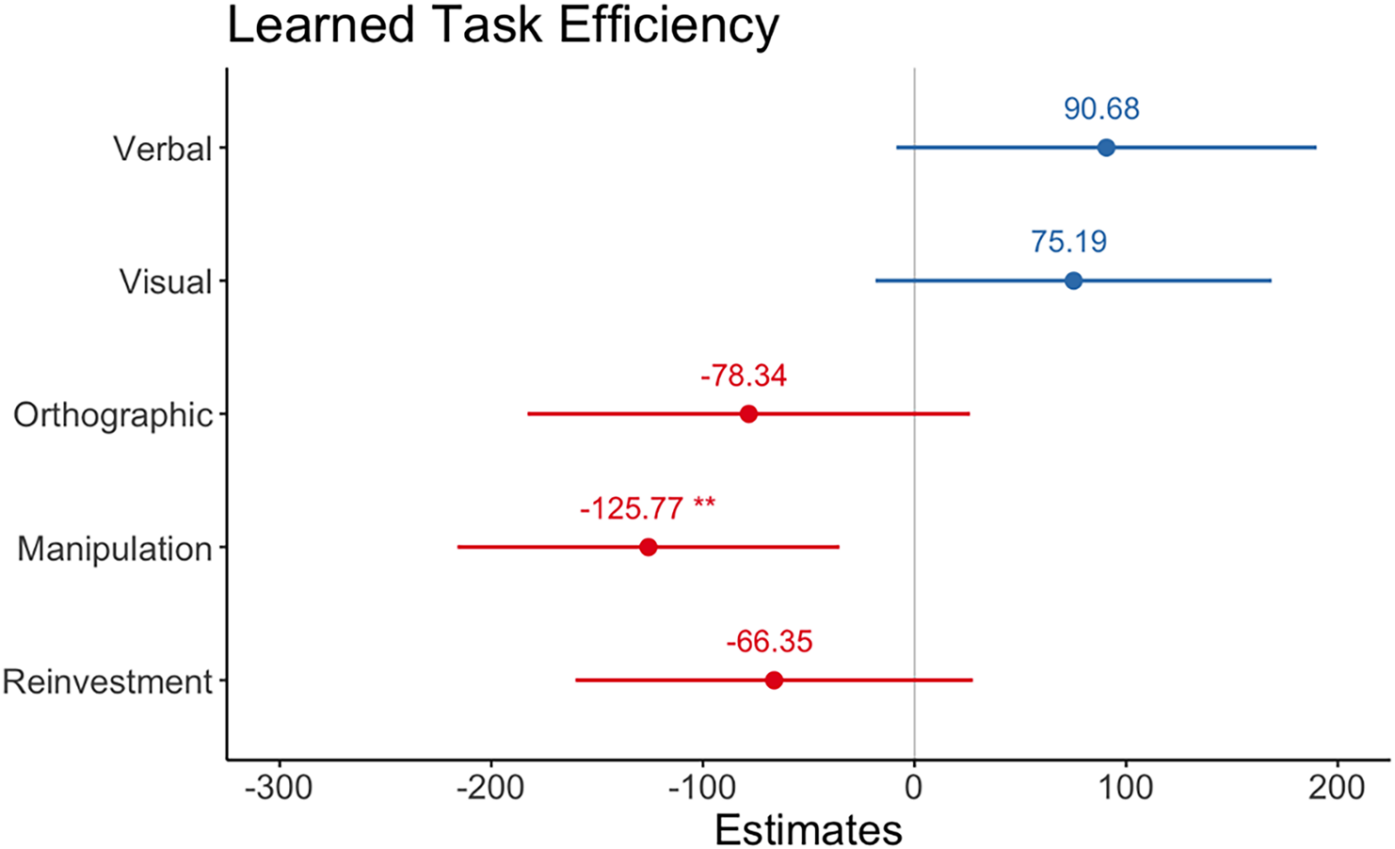
Regression model estimates from linear model for predictors of Learned Task Efficiency. Asterix denotes significant effects. Error bars show 95 percent CI

Consistent with novel performance, degree of Representational Manipulation predicted performance when the task had been learnt, those who reported higher Representational Manipulation were more efficient at performing the task *b* = – 125.77, 95% CI = [– 215.99, – 35.55], *z* = 2.75, *p* = 0.006. No significant effects were observed for Verbal *b* = 90.68, 95% CI = [– 8.63, 0.189.99], *z* = 1.80, *p* = 0.07.), Visual *b* = 75.19, 95% CI = [– 18.43, 168.81], *z* = 1.59, *p* = 0.11 or Orthographic Representations *b* = – 78.34, 95% CI = [– 182.91, 0.26.23], *z* = – 1.39, *p* = 0.16. There was also no significant effect of Total Reinvestment b = 66.35, 95% CI = [– 160.22, 27.91], z = – 1.40, *p* = 0.16 (see Fig. 6).

### IRQ, reinvestment, and novel task jitter

**Fig. 7:**
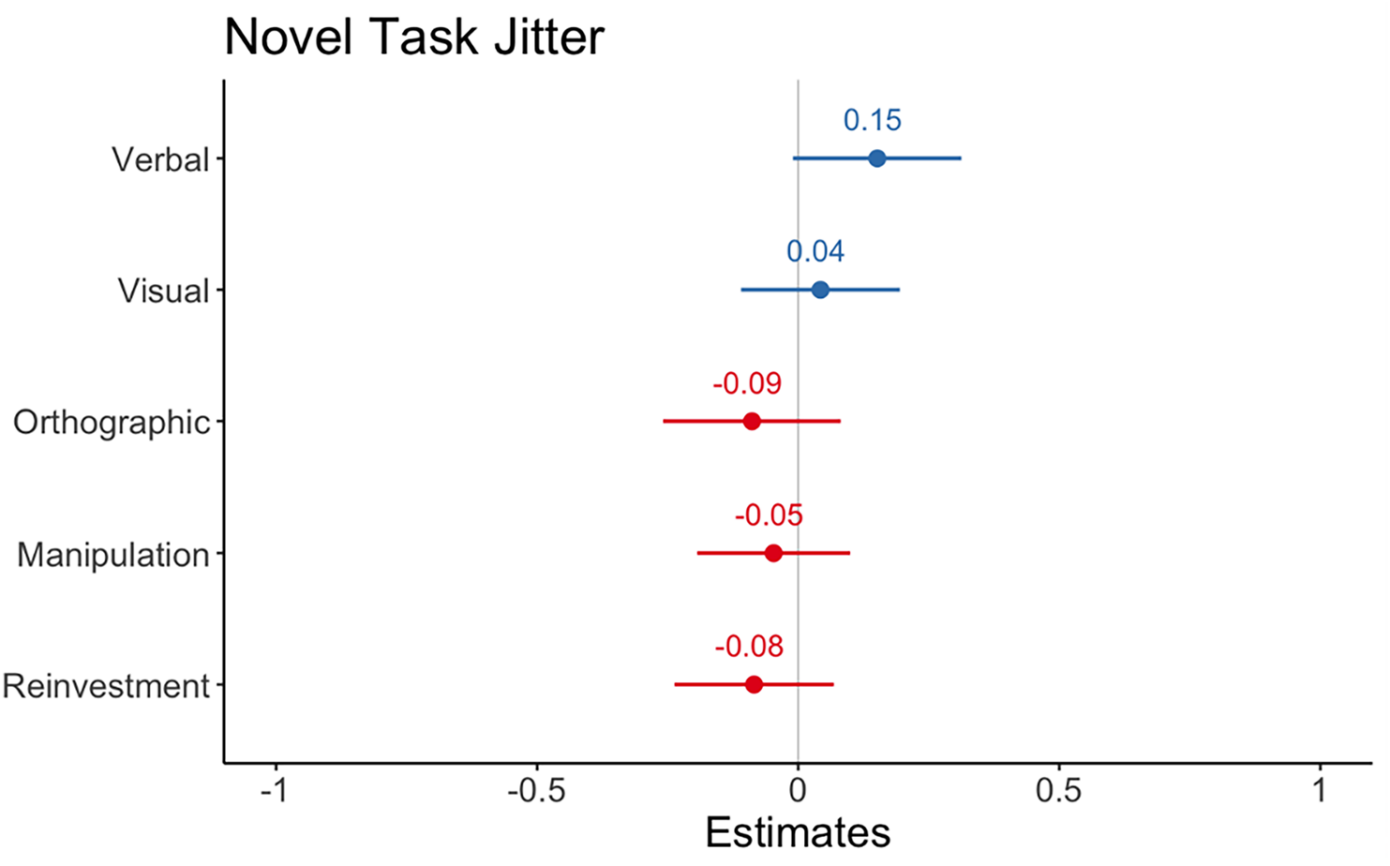
Regression model estimates from linear model for predictors of Novel Task Jitter. Asterix denotes significant effects. Error bars show 95 percent CI

There were no significant effects when the task was novel for any of the predictors; degree of representational manipulation *b* = – 0.047, 95% CI = [– 0.19, 0.10], *z* = – 0.634, *p* = 0.53. Verbal *b* = 0.151, 95% CI = [– 0.00, 0.31], *z* = 1.85, *p* = 0.06., Visual *b* = 0.04, 95% CI = [– 0.11, 0.19], *z* = 0.55, *p* = 0.58, Orthographic Representations *b* = – 09, 95% CI = [– 0.26, 0.08], *z* = – 0.1.03, *p* = 0.30 or Total Reinvestment b = – 0.08, 95% CI = [– 0.23, 0.07], *z* = – 0.1.09, *p* = 0.28 (see Fig. 7).

### IRQ, reinvestment, and learned jitter

**Fig. 8:**
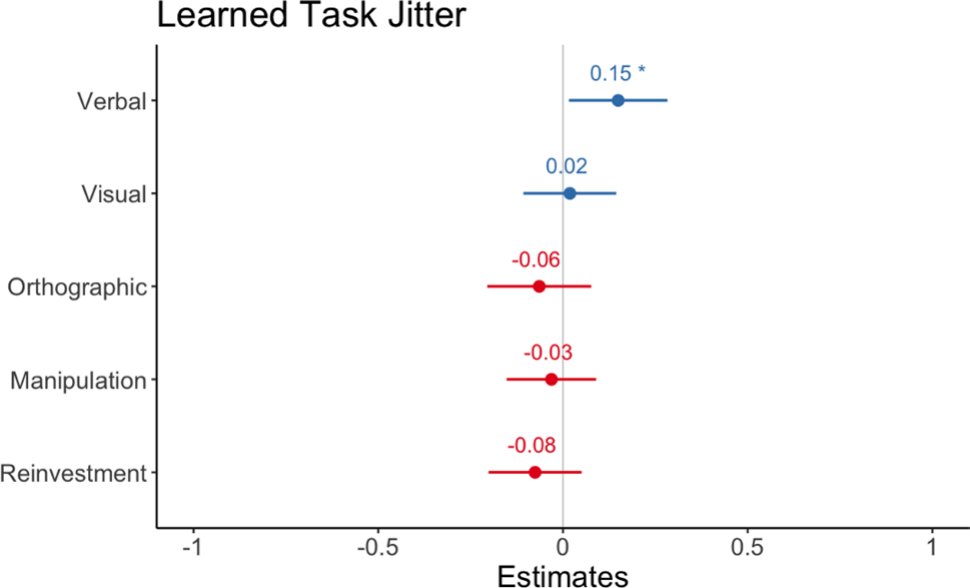
Regression model estimates from linear model for predictors of Learned Task Jitter. Asterix denotes significant effects. Error bars show 95 percent CI

For learned performance, degree of Representational Manipulation did not predict mouse jitter *b* = – 0.03, 95% CI = [– 0.15, 0.09], *z* = – 0.51, *p*  = 0.61. There was a positive relationship with Verbal Representations, such that those with higher verbal representations made mouse responses that were more jittery *b* = 0.149, 95% CI = [0.02, 0.29], *z* = 2.21, *p* = 0.02. Visual *b* = 0.15, 95% CI = [– 0.11, 0.14], *z* = 0.29, *p* = 0.77, Orthographic representations *b* = – 0.06, 95% CI = [– 0.20, 0.08], *z* = – 0.90, *p* = 0.37 and Total Reinvestment *b* = – 07, 95% CI = [– 0.20, 0.05], *z* = – 1.19, *p* = 0.24 were not significant predictors (see Fig. 8).

## Discussion

This study investigated the relationship between internal representations, conscious motor control, and motor performance efficiency and jitter. Participants completed the internal representations questionnaire (IRQ; Roebuck & Lupyan, 2020), movement specific reinvestment scale (MSRS; Masters et al., 2005), and a novel continuous, closed-loop cursor movement task before and after practice, while movement performance efficiency (speed accuracy trade off) and jitter were recorded. Findings showed that the IRQ Verbal factor had significant predictive value for the Total Reinvestment and the MSRS sub-scale of Movement Self Consciousness (MSC) but not Conscious Motor Process (CMP). However, only the IRQ factor of Manipulational Representation was a significant predictor of motor performance, and this occurred both before and after the period of practice. There were no significant predictors of the jitter in cursor movements when the task was novel, however, the IRQ Verbal factor did predict higher levels of jitter after practice.

The first hypothesis was supported by the data. The IRQ Verbal and Orthographic factors had significant positive predictive value for Total Reinvestment as well as sub-scales of for MSC but not CMP. This suggests that, while these two fields of work have developed separately, there is some conceptual overlap between the propensity to use internal verbalisations and potential for reinvestment (Lupyan, 2016; Masters & Maxwell, 2008). This presents a novel development in the literature and suggests broader integration of individual difference in phenomenology and motor control research is called for. Here, we have focused on movement specific reinvestment, but this overlap could extend to domains where the theory of reinvestment has been applied to investigate engagement in conscious control of decision-making and other activities (Kinrade et al., 2015).

Reinvestment did not predict performance of a motor task in this study, but IRQ factors did, both before and after the movement was learned. This did, however, occur for unexpected reasons. It was the Representational Manipulation factor that was the only predictor of performance on the task. We had hypothesized that high reinvestment scores and internal verbalisation would predict poorer performance (Masters & Maxwell, 2008). IRQ Verbal factor did predict higher levels of jitter after the task was learned, suggesting that time spent verbalizing a movement during practice could have a negative effect, but this did not show in overall performance data. Due to the relationship between imagery and performance (Romano-Smith et al., 2018), we expected high IRQ Visual scores to be associated with efficient motor performance (Hardwick et al., 2018). However, it is possible that the representational manipulation factor does also capture an element of imagery.

The IRQ factor of Representational Manipulation refers to the ability to manipulate what is represented in the mind, for example seeing a shape a moving it to a different orientation. Participants with a high propensity for this function may be better able to represent and manipulate the movement aspect of the task. This process could be more closely related to motor imagery, with the IRQ Visual factor relating to imagery more generally. For example, in recent work investigating mental rotation performance, participants with developmental coordination disorder were less able than controls to perform a motor imagery task (involving rotating a hand) but showed no differences in their ability to perform a common visual imagery task without manipulation (Barhoun et al., 2021). Research designs that focus on the relationship between motor imagery, common visual imagery, and manipulational representations as they are defined in the IRQ would be beneficial in establishing how the representations link to other specific types of imagery and how these may then relate to motor control.

Other perspectives on skilled performance have argued against the conceptualisations promoted in the conscious motor processing and reinvestment literature and could explain the benefits of high propensity for manipulational representations (Christensen, 2020; Christensen et al., 2015, 2016; Toner & Moran, 2020). For example, Toner and Moran (2020) argue that skilled performance is a combination of conscious control and automated movements and that performers must be able to consciously engage motor processes to improve at the task. It could be that, to a point, conscious control, and especially the propensity to engage in manipulational representations and, therefore, imagine movement of the cursor in this task, may be a useful strategy that can more efficiently improve the task. Future work that investigates the processes that participants go through during the motor learning process could answer the question on the advantages of manipulational representations.

There are some explanations for verbalisation and reinvestment not being a predictor of performance in this task. Previous work has found that high reinvestors can display poorer performance, but only under pressure. Here, we had a test of performance after the task had been practiced but no anxiety or pressure manipulations or measures. For example, Park et al. (2020) showed that high reinvestors can show enhanced inhibitory control but this is moderated by trait anxiety, with reinvestment only having a negative effect in high anxiety situations. Similarly, Ellmers et al. (2020) showed that conscious motor control did not predict overly cautious gait in elderly participants, but it was the interaction between MSRS scores and inhibition that predicted gait performance. However, it is important to consider the extent to which task findings may be specific to the nature of a particular task, rather than motor control more broadly. The task employed here was a continuous and closed loop precision motor task. The movements (and processing) required here will vary considerably from sports and walking tasks discussed in other conscious motor control and reinvestment literature (Ellmers & Young, 2018; Kinrade et al., 2015). We anticipate that in our task, there was less possibility to verbalise and reinvest where there are relatively few movements of the arm, hand and fingers that can be consciously controlled compared to the relative complexity of gait or sports skills. For example, “place fingers, moved arm away, sweeping up and over, click”, for this task versus, “feet shoulder width apart, weight central, grip in left palm, overlay right hand, grip loosely, extend back using shoulders and chest, follow through in line with target,” for a golf putt. This supports the arguments of Ranganathan et al. (2021) who suggest that large task variation creates a fragmentation in the motor learning literature and many findings are likely to be highly task specific. It would, therefore, be valuable to use the IRQ in the context of other motor control tasks with varying demands or for others to adopt the use of this cursor movement task.

 The findings of this study present both theoretical and practical implications. Firstly, the conceptual overlap between internal verbalisations and propensity for reinvestment means that researchers in these two fields have potential to expand and exchange concepts and ideas to move both forward. Furthermore, the finding that manipulational representations may be important to the performance motor skills is novel and opens a new route for investigating how representations can predict future motor performance. Future work can build on the initial findings presented here to assess the viability for using the IRQ in a variety of applied settings where predicting motor control processes and subsequent performance can have significant impact. It would be beneficial for this work to attempt to develop better measures of representations during performance and, potentially, during the learning process, to further unpack the value of a propensity for manipulating representations. Finally, this study has shown that investigations into motor control processes are possible using online research designs, and show that measures such as remote mouse tracking, which can collect larger samples more efficiently, are viable in this field (Tsay et al., 2021).

The findings here should also be considered alongside the limitations of the study. The development and use of a novel motor control task did allow for research to be conducted remotely and online, but the abstract nature of this task meant step-by-step instructions were required, which could in turn influence representations of the task. While performance did improve from novel to learned, the online nature of the design created issues with keeping attention. Participants were, therefore, only given access to a short practice period. Participants were clearly informed when their performance was being measured and when it was practice, but anxiety was not deliberately manipulated or measured. A clearer anxiety condition may have been beneficial in testing predictions of reinvestment theory (Park et al., 2020).

This study was the first to investigate the relationships between the propensity to engage in multiple forms of internal representation, measured through the IRQ, conscious control during movement execution, and overall efficiency of motor performance. Findings suggest that there is conceptual overlap between research investigating reinvestment and conscious motor control and the investigation of internal verbalisations in the field of phenomenology. Furthermore, while findings did not support predictions that propensity for internal verbalisation could lead to poor performance through conscious control, it did lead to more jitter in the movement. By including multiple measures of internal representations beyond verbalisation and visualisation, this study uncovered the novel finding that a propensity to engage in manipulational representations can positively affect motor performance. Together, findings suggest that the IRQ could prove fruitful in predicting motor performance and could have applications in a variety of domains.

## Data Availability

The data associated with this project can be found at https://osf.io/kpbm9/?view_only=27f251adb982429e9697d12cb9c58b76
